# Identifying prognostic markers in spatially heterogeneous breast cancer microenvironment

**DOI:** 10.1186/s12967-023-04395-x

**Published:** 2023-08-29

**Authors:** Guohong Liu, Liping Wang, Lili Ji, Dan He, Lihua Zeng, Guangzheng Zhuo, Qian Zhang, Dujuan Wang, Yunbao Pan

**Affiliations:** 1 Department of Radiology, Zhongnan Hospital of Wuhan University, Wuhan University, No.169 Donghu Road, Wuchang District, Wuhan, 430071 China; 2 Department of Laboratory Medicine, Zhongnan Hospital of Wuhan University, Wuhan University, No.169 Donghu Road, Wuchang District, Wuhan, 430071 China; 3 Department of Clinical Pathology, Houjie Hospital of Dongguan, The Affiliated Houjie Hospital of Guangdong Medical University, No.21 Hetian Road, Houjie Town, Dongguan, 523000 China

**Keywords:** Breast cancer, Tumor microenvironment, Immune infiltration, Metabolic marker, Immunological checkpoints, Digital spatial profiling

## Abstract

To gain deeper insights into the microenvironment of breast cancer, we utilized GeoMx Digital Spatial Profiling (DSP) technology to analyze transcripts from 107 regions of interest in 65 untreated breast cancer tissue samples. Our study revealed spatial heterogeneity in the expression of marker genes in tumor cell enriched, immune cell enriched, and normal epithelial areas. We evaluated a total of 55 prognostic markers in tumor cell enriched regions and 15 in immune cell enriched regions, identifying that tumor cell enriched regions had higher levels of follicular helper T cells, resting dendritic cells, and plasma cells than immune cell enriched regions, while the levels of resting CD4 memory in T cells and regulatory (Treg) T cells were lower. Additionally, we analyzed the heterogeneity of HLA gene families, immunological checkpoints, and metabolic genes in these areas. Through univariate Cox analysis, we identified 5 prognosis-related metabolic genes. Furthermore, we conducted immunostaining experiments, including EMILIN2, SURF4, and LYPLA1, to verify our findings. Our investigation into the spatial heterogeneity of the breast cancer tumor environment has led to the discovery of specific diagnostic and prognostic markers in breast cancer.

**Supplementary Information:**

The online version contains supplementary material available at 10.1186/s12967-023-04395-x.

## Introduction

Breast cancer is the most prevalent and lethal malignant tumor in women, representing a significant global threat to their life and health. Traditional clinical and pathological staging of breast cancer, along with molecular biological characteristics, offers clinical criteria for prognostic evaluation of breast cancer patients [1]. Nevertheless, the molecular subtypes based on immunohistochemical analysis and intrinsic subtypes based on genomic analysis have certain limitations, and neither can completely explain the clinical heterogeneity of breast cancer prognosis and treatment. The intratumoral heterogeneity theory diverges from the classification theory of breast cancer [2]. Heterogeneity theory implies the existence of multiple subtypes (spatial heterogeneity) within the same tumor and the ability of tumors to transform between different phenotypes at different stages (temporal heterogeneity). Such heterogeneities are driven by tumor cell state heterogeneity and clonal evolution, as well as by the tumor microenvironment and metabolic reprogramming.

The tumor microenvironment (TME) is a complex network system composed of various cells and cytokines that exhibit high heterogeneity. This heterogeneity contributes to different treatment responses and clinical outcomes in cancer patients [3], posing a significant obstacle to the realization of precision medicine in breast cancer. Tumor-infiltrating immune cells are primary cells in TME that play a dual role in anti-tumor or pro-tumor activities. Numerous studies have shown that the immune-suppressive TME significantly affects the clinical efficacy of immunotherapy and other cancer treatments [4]. The intricate interplay between cancer cells and the tumor immune microenvironment (TIME) is a crucial feature of cancer. Tumors can exert immunosuppressive signals, evade immune recognition, or promote pro-tumor inflammation, thereby affecting the immune microenvironment and driving cancer progression [5]. Mechanisms of immune evasion include weakened immune surveillance, downregulation of co-stimulatory molecules, and/or overexpression of co-inhibitory molecules, which reduce the activity of CD8 + T cells. Immune evasion is a critical condition for breast cancer progression and a key step in the transition from non-invasive to potentially lethal invasive diseases [6]. Further exploration of intratumor heterogeneity can help elucidate the biological mechanisms underlying breast cancer behavior and contribute to the development of reliable prognostic and predictive molecular markers for breast cancer.

There is substantial evidence showing a noteworthy association between breast cancer progression and prognosis with metabolic alterations [7]. One example is that tumor cells require higher glucose uptake efficiency to generate energy and meet the demands of rapid proliferation [7]. Additionally, the presence of ample adipose tissue in breast tissue contributes to abnormalities in lipid metabolism, which increases the risk of breast cancer recurrence and metastasis [8, 9]. Thus, exploring metabolism-related genes can offer novel insights and approaches for the clinical diagnosis and treatment of breast cancer.

The heterogeneity of tumors plays a significant role in disease progression. Identification of the molecular drivers of heterogeneity, particularly in the TME will guide future diagnostic and therapeutic strategies. In this study, we employed GeoMx Digital Spatial Profiling (DSP) technology to conduct spatial transcriptomic sequencing on different regions of clinical breast cancer tissue. Our goal was to investigate the spatial expression patterns of genes and explore the interactions between tumor cells and immune cells in the TME. The objective was to reveal the spatial heterogeneity of gene expression patterns and their clinical significance in the TME, and to provide new strategies for prognostic evaluation and immune therapy of breast cancer.

## Methods

### Patient sample collection

Paraffin-embedded tumor tissue samples were obtained from 125 breast cancer patients who underwent initial surgical operation without prior treatment at the Houjie Hospital of Dongguan, Affiliated Houjie Hospital of Guangdong Medical University. The Ethics and Scientific Committee of the Houjie Hospital of Dongguan, Affiliated Houjie Hospital of Guangdong Medical University approved this study (2021002). A tissue microarray (TMA) was constructed using a TMArrayer (Pathology Devices) to obtain 1.5 mm^2^ tissue cores from 125 patients. We acknowledged that the GeoMx Digital Spatial Profiler has spatial limitations in selecting regions of interest (ROI) within a certain range and may not cover the entire tissue chip. Additionally, we experienced some technical challenges during the experimental process, resulting in the loss of a few cores. As a result, we were able to obtain 65 relatively intact cores for further analysis. Subsequently, 65 cores comprising 107 regions of interest (65 regions enriched with tumor cells, 36 regions enriched with immune cells, and 6 regions enriched with normal epithelial cells) were subjected to DSP RNA assays.

For the immunohistochemistry analysis, 125 instances of breast cancer tissue were included in the study. However, 26 cores were excluded from EMILIN2 analysis, 23 cores were excluded from LYPLA1 analysis, and 28 cores were excluded from SURF4 analysis due to loss of tissue integrity during staining.

### Digital spatial profiling and analysis

The NanoString GeoMx DSP RNA assays were conducted at CapitalBio Technology (Beijing, China) according to the recommended procedure as previously described [10]. To distinguish between different morphologies, we used the GeoMx Solid Tumor TME Morphology Kit (Nanostring, Cat#GMX-RNA-MORPH-HST-12) to stain the tissue. PanCK was used to positively stain epithelial cells, CD45 for immune cells, and SYTO13 for nuclear staining. The pathologist could distinguish healthy epithelial cells from malignant ones based on histological morphology. Regions of interest (ROIs) were selected and assessed by pathologist, and then illuminated using UV light. The indexing oligonucleotides released from each ROI were collected and deposited into designated wells on a microtiter plate. The DSP assay sequencing data were processed using the GeoMx NGS Pipeline (DND). After sequencing, the reads were trimmed, merged, and aligned to a list of indexing oligos to identify the source probe. The unique molecular identifier (UMI) region of each read was used to remove PCR duplicates and duplicate reads, thus converting reads into digital counts. The limit of quantitation (LOQ) was estimated as the geometric mean of the negative control probes plus two geometric standard deviations of the negative control probes. Targets that consistently fell below the LOQ were removed, and the datasets were normalized using upper quartile (Q3) normalization. We conducted principal component analysis using the prcomp function from the gene expression matrix and plotted it with the scatterplot3d package.

### Differential expression and enrichment analysis

The Mann-Whitney U test was employed to compare two groups. Genes were considered significant if they had a fold change of at least 1.5 and a p-value less than 0.05. To conduct gene ontology (GO) enrichment and KEGG enrichment analysis of differentially expressed genes (DEGs), EnrichProfiler R-packages were used, with Benjamini-Hochberg multiple testing correction.

### Weighted correlation network analysis (WGCNA) and protein-protein interaction (PPI) networks of differentially expressed genes

The WGCNA and limma software were employed to conduct a weighted correlation network analysis (WGCNA) on differentially expressed genes (DEGs) from 65 PanCK-expressing and 36 CD45-expressing areas. A Pearson correlation coefficient matrix was constructed from the gene expression matrix, and an adjacency matrix was generated using the optimal power value of the Pearson correlation coefficient matrix. To perform hierarchical clustering by module, a topology overlap matrix (TOM) was constructed, and the clustered modules were automatically separated and merged using the cutree Dynamic function. Both turquoise and blue module genes were subjected to functional enrichment analysis, including gene ontology (GO) and KEGG, using R packages such as clusterProfiler, enrichplot, org.Hs.eg.db, and ggplot2. Genes from the turquoise module and the blue module were separately uploaded to the STRING database (http://www.string-db.org/), with network type (complete STRING network), meaning of network edges (confidence), and minimum interaction score parameters being set. The protein interaction data was input into the Cytoscape software using the STRING database, and the core genes in the protein-protein interaction (PPI) network were selected using the cytoHubba plugin.

### Prognostic gene screening

Utilizing a univariate Cox analysis, 55 genes linked to prognosis were sought in the turquoise module extracted from PanCK-expressing regions, while 15 genes associated with prognosis were investigated in the blue module derived from CD45-expressing regions. The surv cutpoint and surv categorize algorithms were employed to determine the optimal cutoff value for each gene, which was then utilized to categorize gene expression. A log-rank test was conducted to analyze survival rates after categorization. The entire procedure was carried out using R packages survival and survminer.

### Immune infiltration analysis

Using the CIBERSORT algorithm, the proportion of 22 immune cell types was quantified in each of the 107 ROIs (65 PanCK-expressing, 36 CD45-expressing, and 6 NC). Differences in immune cell levels among the three regions were assessed using the Kruskal-Wallis test and the reshape2 and ggpubr packages. A gene set related to immunological function was identified based on previous studies [11]. Single-sample Gene Set Enrichment Analysis (GSEA) was performed using the GSVA, limma, and GSEABase packages to determine the immune function scores for each ROI based on the immune function-related gene set. The Wilcoxon test was used with the limma, ggplot2, and ggpubr tools to compare the scores between the three regions. Correlation analysis of immune cell contents between PanCK-expressing and CD45-expressing areas was conducted independently using the corrplot program and the Spearman technique. The surv cutpoint and surv categorize functions were used to determine the cutoff values for the 22 immune cell contents in PanCK-expressing regions, which were then used to classify the immune cell contents. The limma, survival, and survminer packages were employed along with the log-rank test (P < 0.05) to conduct a survival analysis of immune cells in PanCK-expressing regions. Similarly, the immune function scores were categorized based on cutoff values, and the survival analyses of immune cells in both PanCK- and CD45-expressing regions were carried out using the limma, survival, and survminer packages with the log-rank test.

### HLA expression and immune checkpoint analysis

The levels of HLA gene expression in the three areas were assessed using the Kruskal-Wallis test with the aid of the limma, reshape2, ggplot2, and ggpubr packages. The divergent expression of immune checkpoint-related genes across the three areas was investigated through the Wilcoxon test using the limma, ggplot2, and ggpubr packages. A correlation analysis was conducted between immune checkpoint gene expression and immune cell levels in PanCK and CD45-expressing areas using the Spearman correlation coefficient and the limma, reshape2, tidyverse, and ggplot2 packages. The findings were presented in a visual format.

### Analysis of metabolism-related genes

Based on previous literature, we identified 944 genes involved in metabolic processes [12]. The Gene Set Enrichment Analysis (GSEA) website (http://www.gsea-msigdb.org/gsea/downloads.jsp) provided the “c2.cp.kegg.v7.5.1.symbols.gmt” file, which was used to perform GSVA on the ROIs using the GSEABase, GSVA, limma, and pheatmap packages. Only 20 pathways were selected, and the parameters were set to P < 0.05. A Venn diagram was created by intersecting the 944 metabolic genes with 3515 differential genes (PanCK- versus CD45-expressing areas) obtained from the Bioinformatics website (http://bioinformatics.psb.ugent.be/webtools/Venn/), resulting in 182 genes. Univariate Cox analysis was conducted on the 182 metabolic genes in the 65 PanCK-expressing ROIs to identify genes associated with prognosis, followed by survival analysis and ROC curve plotting using the Survivor, Survminer, and timeROC packages in R.

### Immunohistochemistry

The TMA sections were incubated overnight with anti-human EMILIN2 (Proteintech, 24779-1-AP), SURF4 (Proteintech, 11599-1-AP), or LYPLA1 (Proteintech, 16055-1-AP) antibodies, followed by incubation with a secondary antibody and then subjected to the liquid DAB substrate-chromogen system. The experiments were conducted in accordance with the clinical pathological protocols of the Affiliated Houjie Hospital of Guangdong Medical University.

## Results

### Differential expression and enrichment analysis

**Fig. 1:**
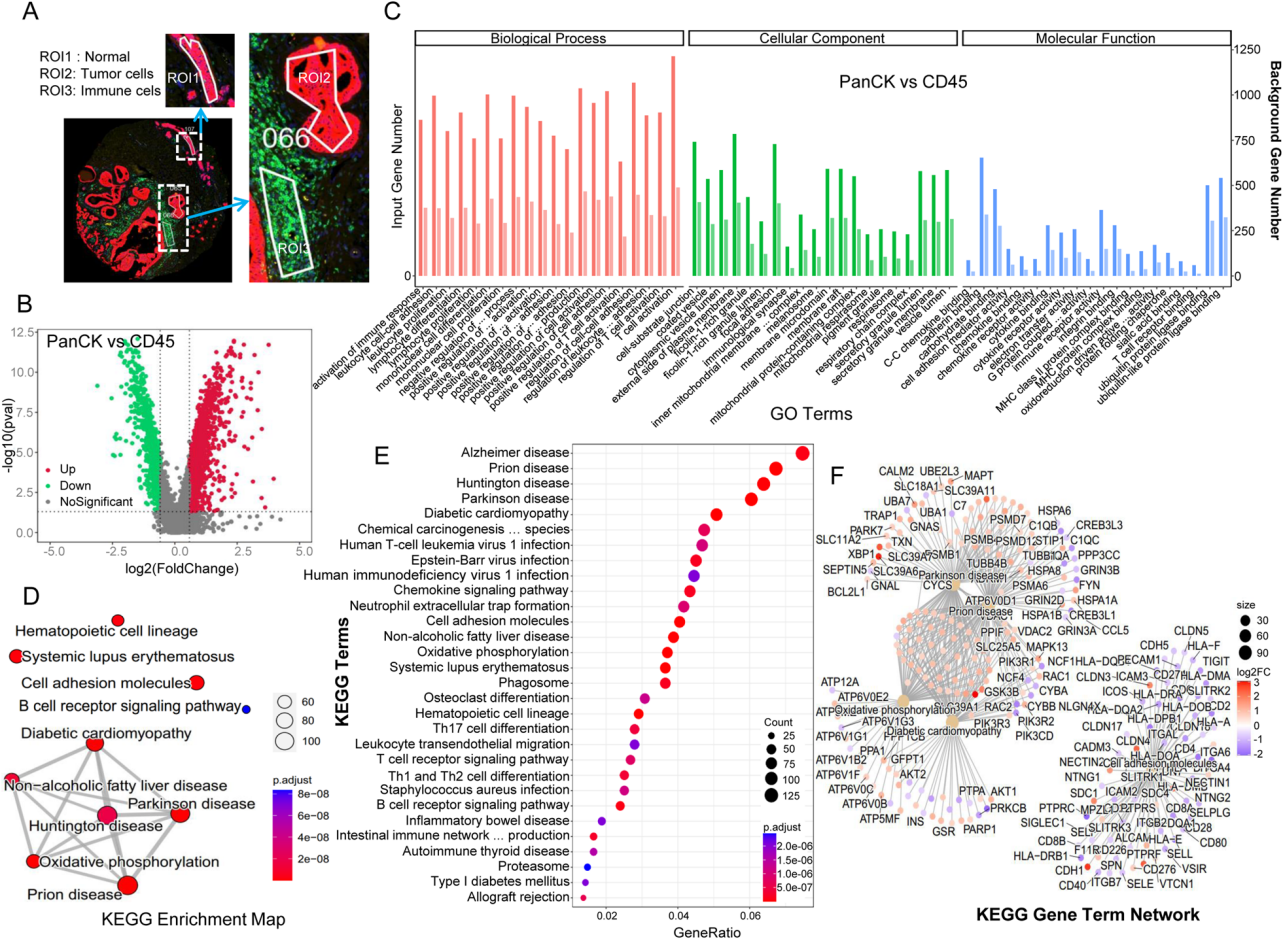
Differential expression and enrichment analysis. **A** regions of interest (ROIs) were selected, including tumor cell (PanCK-expressing), immune cell (CD45-expressing), and normal epithelial cells regions (normal tissue morphology). **B** differential analysis was performed between the tumor cell-enriched regions and immune cell-enriched regions. The upregulated genes are shown in red, while the downregulated genes are shown in green. **C** GO enrichment analysis of differentially expressed genes. (**D**–**F**) KEGG enrichment analysis of differentially expressed genes. PanCK indicates tumor cell-enriched regions, while CD45 indicates immune cell-enriched regions

Fluorescent anti-PanCK and anti-CD45 antibodies were employed to identify the 107 regions of interest (ROIs) in 65 patients with breast cancer. The pathologic morphology of PanCK-expressing ROIs allowed us to differentiate between normal epithelial cells and tumor cells. Consequently, 65 ROIs represented tumor cells (PanCK-expressing), 36 ROIs represented immune cells (CD45-expressing), and 6 ROIs represented normal epithelial cells (NC), as shown in Fig. 1A. The number of regions exhibiting differential gene expression was 3515 (PanCK- vs. CD45-expressing regions), as demonstrated in Fig. 1B. GO and KEGG enrichment analyses were used to investigate these differentially expressed genes (DEGs). The 3515 DEGs were mostly associated with cell-substrate junction, external side of the plasma membrane, and focal adhesion. They exhibited various functions, including cadherin-binding and immune receptor activity, and were involved in biological processes such as activation of immune response and T cell activation. Furthermore, they regulated pathways like oxidative phosphorylation, B cell receptor signaling pathway, and chemokine signaling pathway (Fig. 1D, E, and F).

### Analysis of regional core prognostic genes using WGCNA and PPI networks

**Fig. 2:**
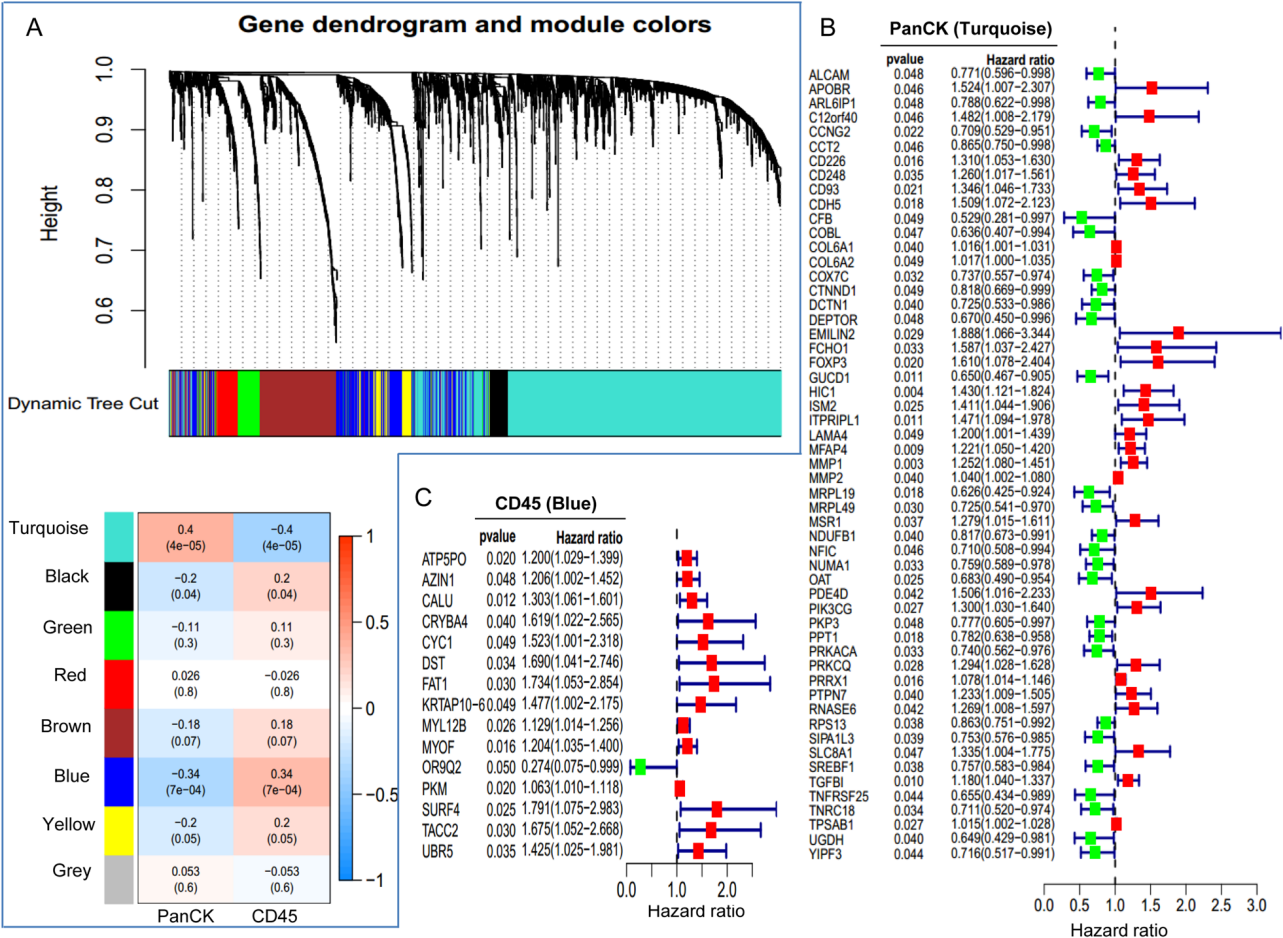
Screening of prognostic genes. **A** Weighted Gene Co-expression Network Analysis (WGCNA). **B** Genes in the turquoise module were subjected to univariate Cox analysis in tumor cell-enriched (PanCK-expressing) regions. **C** Genes in the blue module were subjected to univariate Cox analysis in immune cell-enriched (CD45-expressing) regions. PanCK: regions enriched with tumor cells. CD45: regions enriched with immune cells

**Fig. 3:**
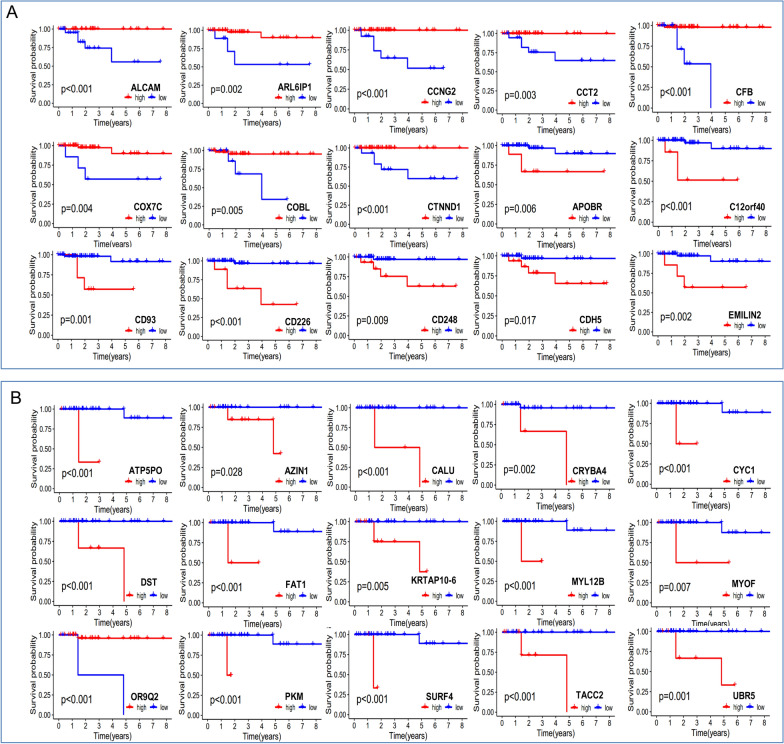
Survival analysis of prognostic genes. **A** Survival analysis of genes in the turquoise module in tumor cell enriched regions (PanCK-expressing). **B** Survival analysis of genes in the blue module in immune cell enriched regions (CD45-expressing). The p values were calculated using the log-rank test

We conducted WGCNA analysis on 3515 DEGs identified between 65 PanCK-expressing and 36 CD45-expressing regions, resulting in the generation of 8 modules following hierarchical clustering. The turquoise module genes exhibited a strong positive correlation with PanCK expression (r = 0.4 and P = 4e-05), while the blue module was significantly positively associated with CD45 expression (r = 0.34 and P = 7e − 04) (Fig. 2A). Based on univariate Cox analysis, we identified 55 genes in the turquoise module linked to PanCK-expressing regions and 15 genes in the blue module linked to CD45-expressing regions that were significantly associated with patient prognosis (P < 0.05) (Fig. 2B, C). Among the genes behaving as protective factors in PanCK-expressing areas were *ALCAM, ARL6IP1, CCNG2, CCT2, CFB, COBL, COX7C, CTNND1, DCTN1, DEPTOR, GUCD1, MRPL19, MRPL49, NDUFB1, NFIC, NUMA1, OAT, PKP3, PPT1, PRKACA, RPS13, SIPA1L3, SREBF1, TNFRSF25, TNRC18, UGDH, and YIPF3, while APOBR, C12orf40, CD226, CD248, CD93, CDH5, COL6A1, COL6A2, EMILIN2, FCHO1, FOXP3, HIC1, ISM2, ITPRIPL1, LAMA4, MFAP4, MMP1, MMP2, MSR1, PDE4D, PIK3CG, PRKCQ, PRRX1, PTPN7, RNASE6, SLC8A1, TGFBI, and TPSAB1* acted as risk factors. Higher expression of the protective factors was associated with better prognosis for breast cancer patients, whereas higher expression of risk factors was associated with poorer prognosis (Fig. 3A and Additional file 1: Fig. S1). For the 15 genes in the blue module associated with CD45-expressing regions, higher expression of *ATP5PO, AZIN1, CALU, CRYBA4, CYC1, DST, FAT1, KRTAP10-6, MYL12B, MYOF, OR9Q2, PKM, SURF4, TACC2, and UBR5* was linked to a worse prognosis, whereas higher expression of OR9Q2 was linked to a better prognosis in breast cancer patients (Fig. 3B).

### Immune infiltration of tumor tissue

**Fig. 4:**
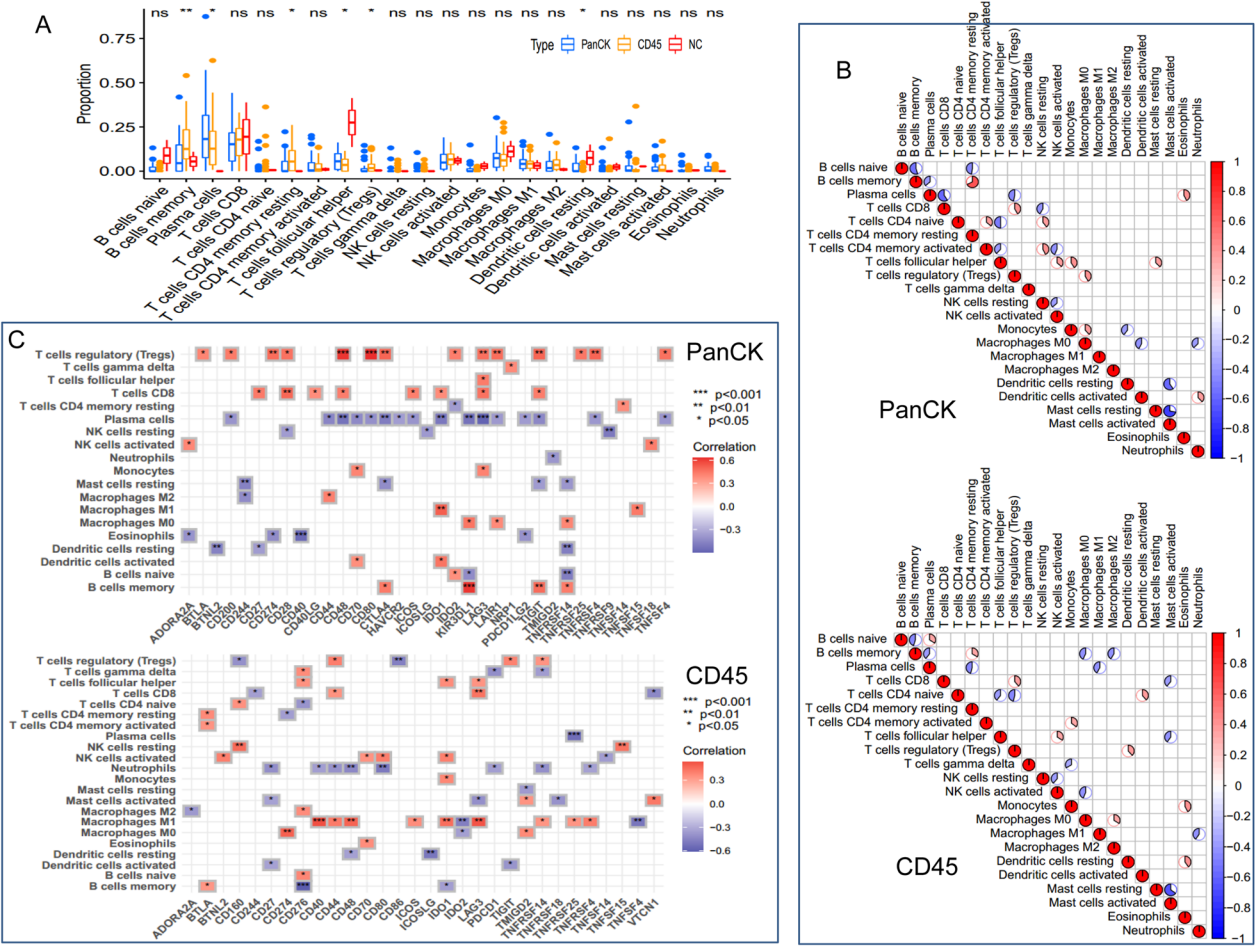
Immune infiltration analysis. **A** Comparison of immune cell levels among the three regions. **B** Correlation analysis between immune cells in PanCK-expressing (tumor cell enriched) and CD45-expressing (immune cell enriched) regions. **C** Correlation analysis between immune cells and immune checkpoints in PanCK- and CD45-expressing regions. PanCK: regions enriched with tumor cells. CD45: regions enriched with immune cells. NC: regions enriched with normal epithelial cells

The relative levels of 22 immune cells in 107 ROIs are shown in Fig. 4A. Differences in B memory cells, plasma cells, T cells CD4 memory resting, follicular helper T cells, Tregs, and dendritic cells resting were observed among the three groups. As shown in Fig. 4B, the correlation of each type of immune cell differed between regions. In regions where PanCK was expressed, follicular helper T cells had negative correlations with resting CD4 naïve T cells and CD4 memory activated T cells, but positive correlations with activated NK cells, monocytes, and resting mast cells. Conversely, in regions where CD45 was expressed, follicular helper T cells had negative correlations with resting CD4 naïve T cells and activated mast cells, but positive correlations with activated NK cells. In PanCK-expressing areas, Tregs were positively associated with CD48 and CD80, T cells CD8 with CD28, and B cells memory with KIR3DL1. In contrast, negative associations were found between plasma cells and LAG3, NK cells resting and TNFRSF9, mast cells resting and CD244, and eosinophils and CD40 (Fig. 4C, top). In CD45-expressing regions, positive connections were observed between CD8 + T cells and LAG3, NK cells resting and CD160 and TNFSF15, macrophages M1 and CD40, and macrophages M0 and CD274. Plasma cells and TNFRSF25, neutrophils and CD48 and CD80, resting dendritic cells and ICOSLG, and B memory cells and CD276 all showed negative correlations (Fig. 4C, bottom).

**Fig. 5:**
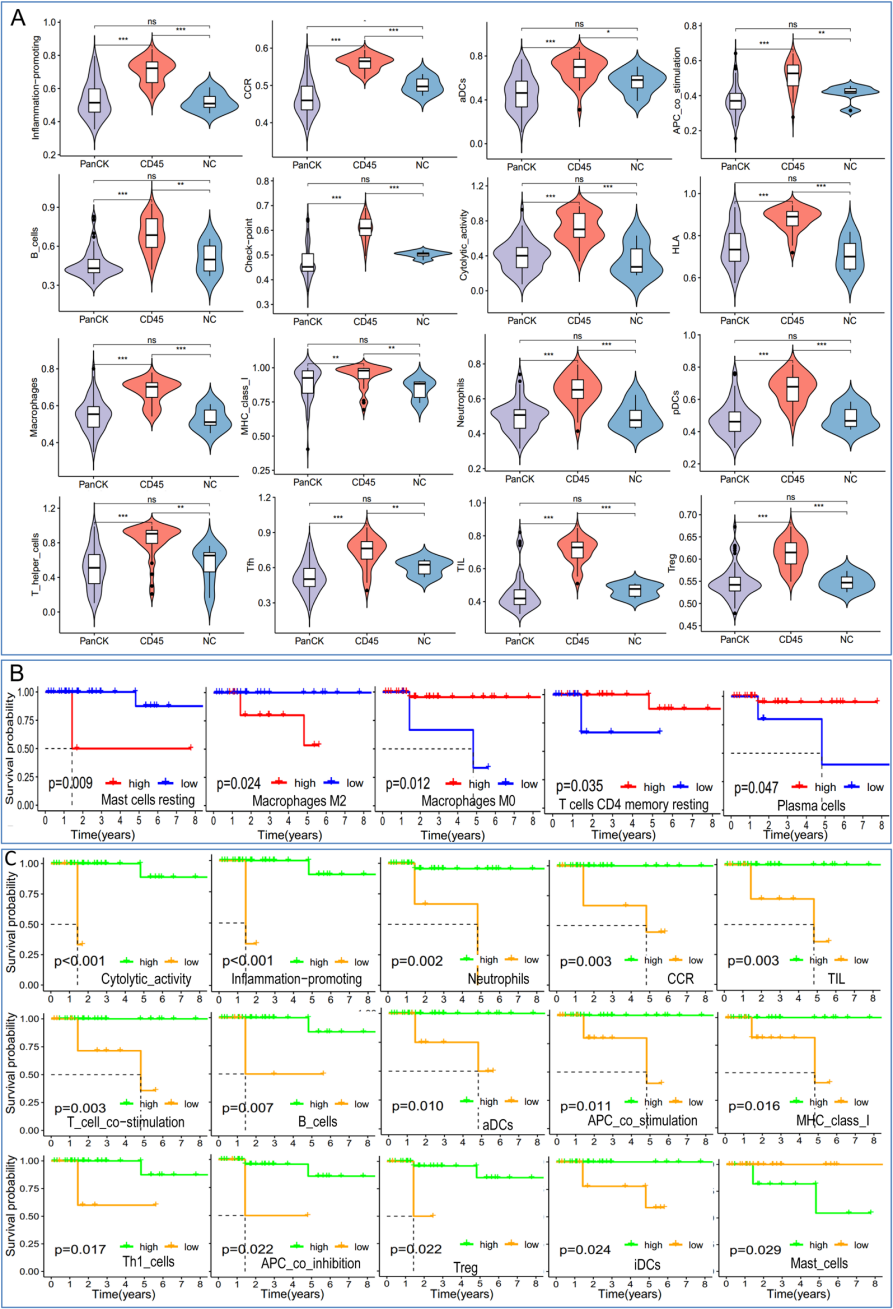
Analysis of immune functions and their effect on survival. **A** Differences in immune functions among three groups: tumor cell enriched (PanCK-expressing), immune cell enriched (CD45-expressing), and normal epithelial cells enriched regions. **B** The effect of immune cell levels on survival in breast cancer patients. **C** The effect of immune functions on survival in breast cancer patients. For the Kaplan Meier curves, the p values were calculated using the log-rank test

The expression of immune cells and their functions varied among different regions (Fig. 5A and Additional file 1: Fig. 2). CD45-expressing areas showed the highest expression of molecules associated with inflammation promotion, CCR, aDCs, APC co-stimulating, B cells, Check-point, Cytolytic activity, HLA, Macrophages, MHC class I, Neutrophils, pDCs, T helper cells, Tfh, TIL, and Treg (Fig. 5A). High levels of resting Mast cells or M2 Macrophages were associated with a poorer prognosis, while an increased presence of M2 Macrophages, T cells CD4 memory resting or Plasma cells were related to a better prognosis (Fig. 5B). The immune function’s survival analysis is depicted in Fig. 5 C.

### HLA and immune checkpoints

**Fig. 6:**
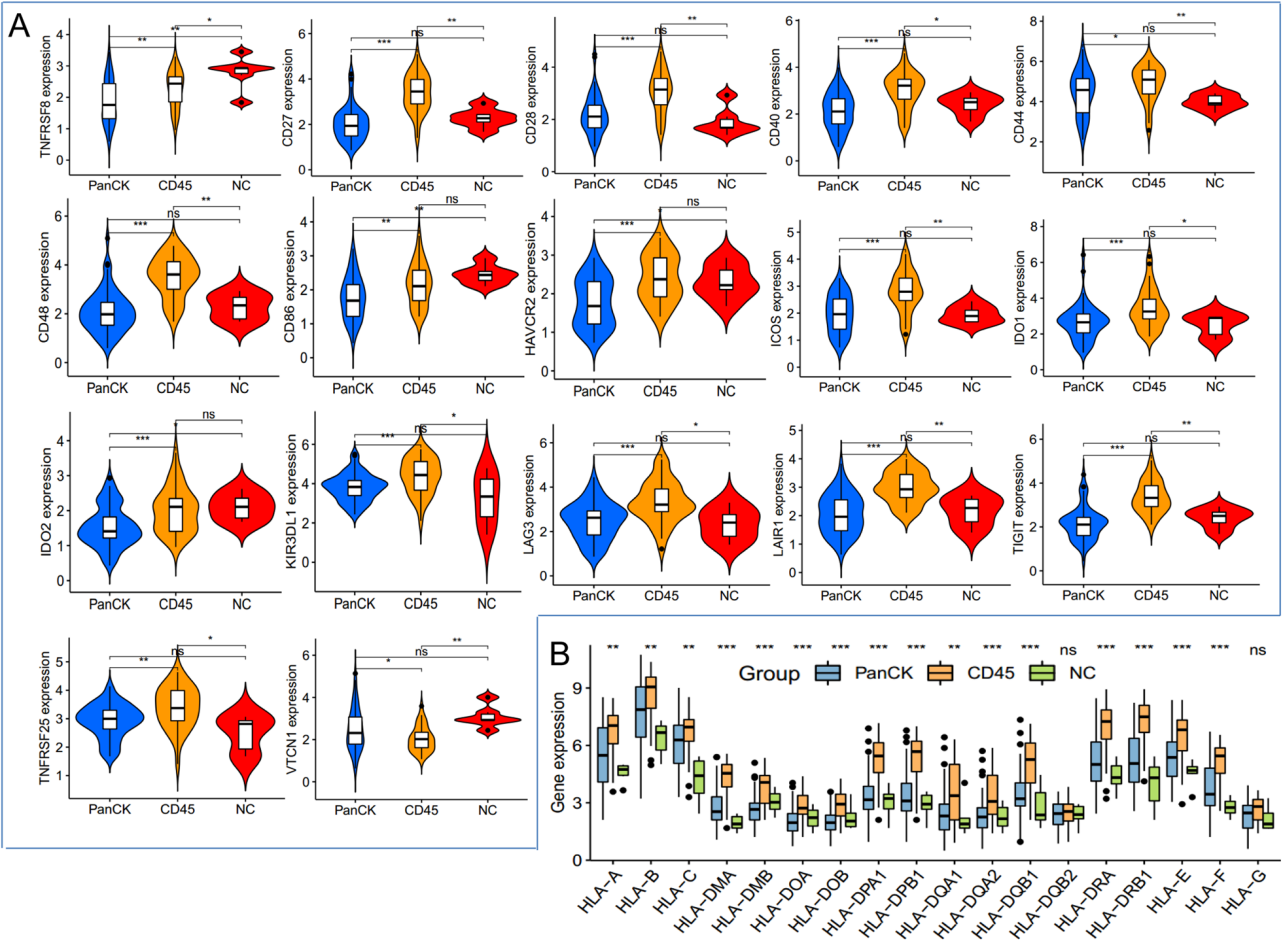
Differential expression of immune checkpoints and HLA genes in three regions. **A** Expression of immune checkpoint-related genes in tumor cell enriched (PanCK-expressing), immune cell enriched (CD45-expressing), and normal epithelial cells enriched regions (NC). **B** Expression of HLA-related genes in the three regions

There were variations in the expression of immune checkpoint-related genes across different regions. CD45-expressing areas exhibited higher levels of expression of *TNFRSF8, CD27, CD28, CD40, CD44, CD48, CD86, HAVCR2, ICOS, IDO1, IDO2, KIR3DL1, LAG3, LAIR1, TIGIT, TNFRSF25, BTLA, BTNL2, CD160, CD200, CD200R1, CD244, CD274, CD40LG, CD70, CD80, CTLA4, ICOSLG, PDCD1, PDCD1LG2, TMIGD2, TNFRSF14, TNFRSF4, TNFRSF9, TNFSF14, TNFSF15, TNFSF18, TNFSF4, and TNFSF9*, compared to PanCK-expressing areas (Fig. 6A and Additional file 1: Fig. S2). Additionally, HLA gene expression varied amongst the three regions, with CD45-expressing areas exhibiting the highest levels of HLA gene expression. Specifically, *HLA-A, HLA-B, HLA-C, HLA-DMA, HLA-DMB, HLA-DPA1, HLA-DPB1, HLA-DQA1, HLA-DQA2, HLA-DQB1, HLA-DRA, HLA-DRB1, HLA-E, and HLA-F* expression were higher in CD45-expressing areas than in PanCK-expressing regions (Fig. 6B).

### Metabolism-related genes

**Fig. 7:**
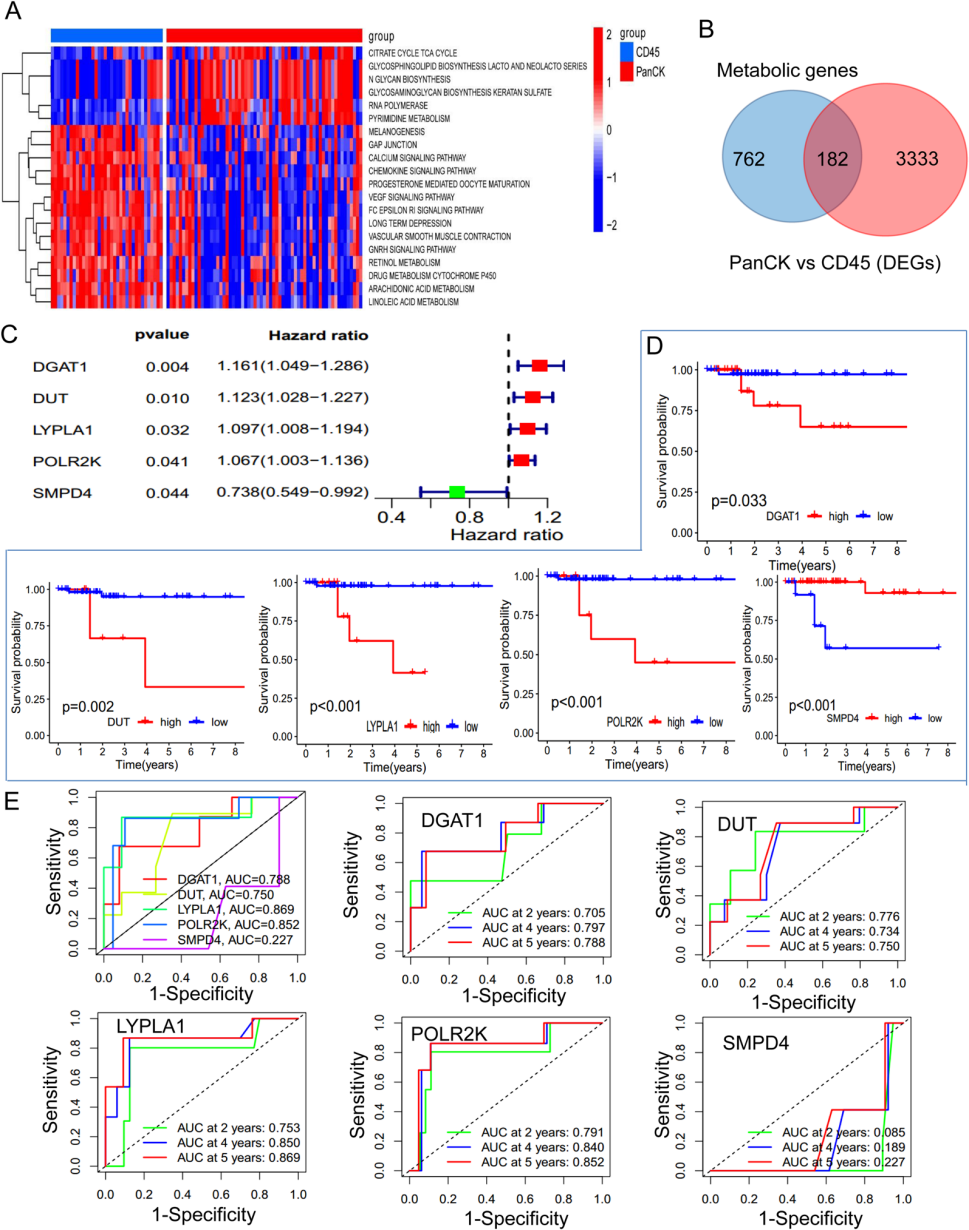
Analysis of metabolism-related genes. **A** GSVA enrichment analysis of PanCK-expressing and CD45-expressing regions. **B** There were 944 metabolic genes, 3515 differentially expressed genes (PanCK-expressing regions vs. CD45-expressing regions), and 182 intersecting genes. **C** Univariate Cox analysis was performed on 134 intersecting genes in PanCK-expressing regions. **D** Survival analysis was conducted on prognostic metabolic genes in PanCK-expressing regions. **E** ROC curves were used to predict the survival rates of breast cancer patients. For the Kaplan Meier curves, the p values were calculated using the log-rank test

The expression of metabolic genes varied between PanCK-expressing and CD45-expressing regions. In PanCK-expressing regions, metabolic genes were primarily involved in citrate cycle TCA cycle, glycosphingolipid biosynthesis LATCO and NEOLATCO series, N glycan biosynthesis, glycosaminoglycan biosynthesis keratan sulfate, RNA polymerase, and pyrimidine metabolism. In contrast, in CD45-expressing regions, metabolic genes were predominantly involved in the calcium signaling pathway, VEGF signaling pathway, FC epsilon RI signaling pathway, vascular smooth muscle contraction, GNRH signaling pathway, retinol metabolism, arachidonic acid metabolism, and linoleic acid metabolism (Fig. 7A). The intersection of 944 metabolic genes and 3515 differential genes from PanCK-expressing and CD45-expressing areas resulted in 182 genes (Fig. 7B). Using univariate Cox regression analysis, five metabolic genes were found to be associated with breast cancer prognosis in PanCK-expressing areas. The expression of *DGAT1, DUT, LYPLA1, and POLR2K* was linked to an increased risk of breast cancer, whereas SMPD4 was associated with a protective effect (Fig. 7C). Higher expression of *DGAT1, DUT, LYPLA1, and POLR2K* in PanCK-expressing regions was linked to a poorer prognosis, whereas a greater expression of SMPD4 was associated with better survival (Fig. 7D). Figure 7E showed the ROC curves for patients’ survival at 2, 4, and 5 years, indicating that *LYPLA1* expression accurately predicted 2-, 4-, and 5-year survival rates in patients with breast cancer (AUC = 0.753, 0.850, and 0.869).

Estrogens signaling pathways play a critical role in the development, progression, and survival of breast cancer. In our study, we categorized the estrogen receptor (ER) expression levels in breast cancer patient tissues and identified differentially expressed genes between the negative and positive groups. In ER-positive tissues, the differentially expressed genes were primarily associated with porphyrin and chlorophyll metabolism, fructose and mannose metabolism, lysosome, and glycosphingolipid biosynthesis globo series. Conversely, in ER-negative tissues, the genes were predominantly associated with primary immunodeficiency, alzheimer’s disease, and pathways in cancer (Additional file 1: Fig. S3A). Through univariate Cox regression analysis, we identified three genes that were significantly associated with breast cancer prognosis in PanCK-expressing areas. The expression of AMOTL1 and IFRD1 was linked to an increased risk of breast cancer, whereas ARRB1 was associated with a protective effect (Additional file 1: Fig. S3B). Higher expression of AMOTL1 and IFRD1 in PanCK-expressing regions correlated with poorer prognosis, while greater expression of ARRB1 was associated with better survival (Additional file 1: Fig. S3C). The ROC curves for patients’ survival at 2, 4, and 5 years demonstrated that the expression of these three genes accurately predicted the 2-, 4-, and 5-year survival rates in patients with breast cancer (Additional file 1: Fig. S3D).

### Validation of protein levels of biomarkers

**Fig. 8:**
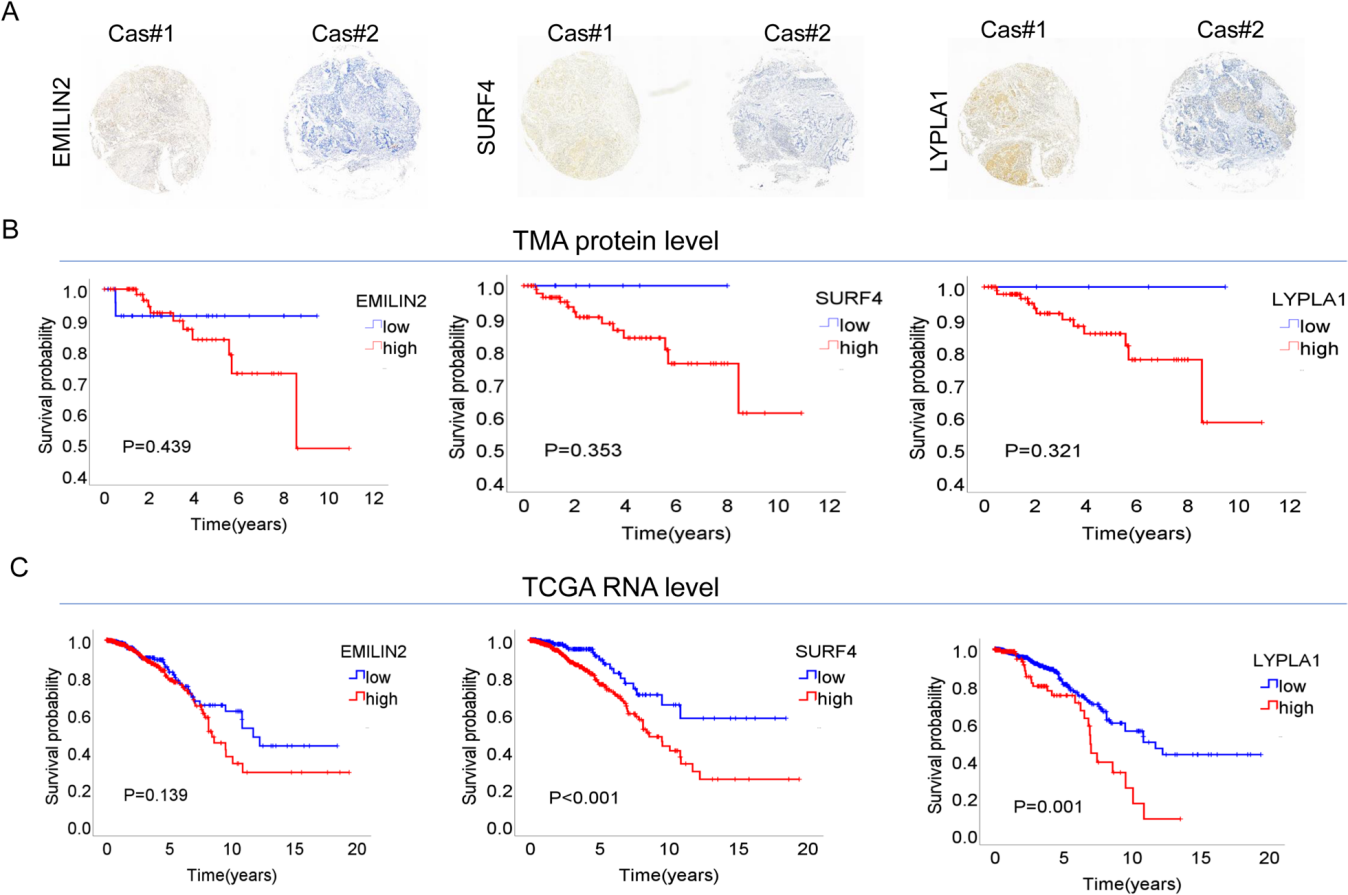
Verification of prognostic genes. **A** Differential protein expression of EMILIN2, SURF4, or LYPLA1 in breast cancer patients, as determined by immunohistochemistry. Representative images of low and high expression are shown. **B** Kaplan-Meier survival curves for breast cancer patients stratified by protein expression levels of EMILIN2, SURF4, or LYPLA1. Patients with higher protein levels have worse overall survival. **C** The bulk RNA sequencing data and clinical information of breast cancer were downloaded from the TCGA database. Subsequently, kaplan-Meier survival analysis were conducted for breast cancer patients. Patients with higher RNA levels have worse overall survival. The p values were calculated using the log-rank test

The characteristics of breast cancer patients from the validation cohort used for immunostaining experiments are described in Additional file 1: Table S1. We conducted immunostaining experiments, including EMILIN2, which showed the most significant prognostic effect in the Pan-CK enriched region, SURF4, which exhibited the most apparent prognostic effect in the CD45 enriched region, and metabolic gene LYPLA1, to verify our findings (Fig. 8A). Consistent with the conclusion based on transcript levels, breast cancer patients with high expression levels of the above-mentioned three proteins had a poorer prognosis (Fig. 8B). In addition, we downloaded the bulk RNA sequencing data and clinical information of breast cancer from the TCGA database. Subsequently, we conducted analysis to validate the above three biomarkers and obtained similar results (Fig. 8C).

## Discussion

Tumor heterogeneity is a significant obstacle in cancer research, as it is a common feature of human tumors. This heterogeneity can be categorized into inter-tumoral heterogeneity and intra-tumoral heterogeneity. The latter can present as temporal heterogeneity, where the molecular composition of cancer cells changes over time, or spatial heterogeneity, where cancer cells exhibit non-uniform distribution in different regions or compartments of the tumor. The identification of tumor heterogeneity has significant implications for tumor diagnosis, treatment, prognosis, and the analysis of molecular biomarkers [13]. However, recognizing tumor heterogeneity is challenging because once cancer cells are isolated in suspension, they lose their positional information. Therefore, the application of spatial transcriptomics is necessary to simultaneously identify cell types within the tumor and their locations in the tissue.

The results of the study are highly relevant to breast cancer research as they provide important insights into the molecular and immune landscape of the tumor microenvironment. By analyzing differential gene expression between PanCK-expressing (tumor cells) and CD45-expressing (immune cells) regions, the study identified distinct sets of genes associated with breast cancer prognosis. These findings shed light on the role of specific genes and immune cell populations in tumor development and patient outcomes.Using DSP technology, we examined 15 prognosis markers in immune cell-enriched regions and 55 prognostic gene markers in tumor cell-enriched regions. The sets of genes identified through WGCNA analysis and univariate Cox regression analysis are particularly noteworthy. The turquoise module genes were positively correlated with PanCK expression. These genes, including *ALCAM*, ARL6IP1 and CCNG2 may play a protective role in breast cancer. On the other hand, the blue module genes showed positive correlation with CD45 expression. Among them, genes like *AZIN1, MYOF*, and *TACC2* were identified as potential risk factors. In regions with a high concentration of tumor cells, we also investigated genes associated with metabolism that could impact the prognosis of breast cancer patients. To validate our findings, we utilized immunohistochemistry to confirm the prognostic effects of EMILIN2 in the Pan-CK enriched region, SURF4 in the CD45 enriched region, and LYPLA1, a metabolic gene. EMILIN2 was identified as an independent prognostic biomarker that could be associated with the malignancy and development of gliomas, with high expression predicting a poor prognosis [14]. High expression of SURF4 was also observed in breast cancer tissue and cells, with SURF4 promoting the proliferation and migration of breast cancer cells and being associated with poor prognosis [15]. LYPLA1 was found to play a tumor-promoting role in non-small cell lung cancer (NSCLC) cells in vitro, with suppression of its expression leading to significant inhibition of proliferation, migration, and invasion of NSCLC cells. Additionally, LYPLA1 was found to be highly expressed in malignant cervical cancer tissues, where it was associated with the upregulation of EMT-inducing TIAM1 and GREM1 and a decrease in mesenchymal markers [16, 17].

The analysis of various tumors through histopathology has confirmed that the composition of infiltrating immune cells within the tumor is distinct from that in the surrounding non-tumor area. The interaction between tumor cells and adjacent stromal cells is an important driving force in promoting tumor progression [18]. The study explored the immune infiltration patterns in different regions of breast cancer tissues. The presence of certain immune cell types, such as M2 Macrophages, was associated with a worse prognosis, while other immune cells like T cells CD4 memory resting and Plasma cells were linked to better patient outcomes. These findings emphasize the crucial role of the immune microenvironment in breast cancer progression and suggest potential targets for immunotherapy. The differential expression of HLA genes and immune checkpoint-related genes in different regions provides additional insights into immune response regulation in breast cancer. CD45-expressing areas exhibited higher expression levels of several immune checkpoint-related genes, indicating potential immunosuppressive mechanisms in these regions. Understanding the intricate interactions between immune cells and tumor cells can inform the development of novel immunotherapies for breast cancer. By targeting specific immune cell populations or modulating immune checkpoint pathways, researchers may be able to enhance the anti-tumor immune response and overcome immunosuppressive mechanisms. This has the potential to revolutionize breast cancer treatment, as immunotherapies have shown remarkable success in various cancer types.

The development and progression of tumors are closely linked to metabolism, and metabolic genes may play a significant role in the prognosis of cancer patients [19]. This study identified metabolic gene sets with distinct activity patterns in PanCK-expressing and CD45-expressing regions. The study’s findings related to metabolic gene activity also offer new perspectives on breast cancer biology. Dysregulated metabolism is a hallmark of cancer, and understanding the specific metabolic pathways involved in breast cancer progression could lead to the development of targeted therapies that disrupt tumor growth and survival [20]. Five genes were found to impact patient survival. DGAT1, a gene known to promote tumor progression in ovarian and prostate cancer [21, 22], was found to promote the proliferation and migration of breast cancer cells [23]. DUT, overexpressed in 42% of HCC tumors, was found to correlate with advanced stage HCC and promote cell cycle arrest and DNA damage. Transcriptome analysis revealed that NF-κB signaling is the downstream effector pathway of DUT, and overexpressing DUT in liver progenitor organoids conferred drug resistance to TKI Sorafenib [24]. LYPLA1 was found to be associated with poor prognosis in lung adenocarcinoma [25], while POLR2K was identified as one of the top 10 cancer immunotherapy proteins related to breast cancer by machine-learning predictions [26]. SMPD4 was also found to be a lipid metabolism-related gene associated with hepatocellular carcinoma prognosis [27]. Overall, this study sheds light on the relationship between metabolism and breast cancer, and provides a basis for further molecular biology experiments. However, additional analysis using larger samples from diverse populations is needed to confirm these findings.

In conclusion, this study enhances our understanding of breast cancer biology, focusing on gene expression, immune infiltration, and metabolic processes. The identified gene sets and immune-related factors have potential as prognostic biomarkers and therapeutic targets for breast cancer patients. These insights into immune modulation and metabolic dysregulation offer novel treatment opportunities that can complement traditional approaches. The findings underscore the importance of personalized treatment approaches that consider the unique molecular and immune characteristics of individual tumors. Overall, this research opens up new avenues for clinical interventions and may improve patient outcomes in breast cancer management.

### Supplementary Information

**Additional file 1: Figure S1.** Survival analysis of prognostic genes.**A** Survival analysis of genes in the turquoise module in tumor cell enriched regions. **B** Survival analysis of genes in the blue module in immune cell enriched regions. **Figure S2.** Differential expression of immune checkpoint-related genes among the three regions. Data shows the expression of immune checkpoint-related genes in tumor cell (PanCK), immune cell (CD45), and normal epithelial cells enriched regions (NC). **Figure S3.** Analysis of ER-related genes. **A** GSVA enrichment analysis of PanCK-expressing and CD45-expressing regions. **B** There were 944 metabolic genes, 3515 differentially expressed genes (PanCK-expressing regions vs. CD45-expressing regions), and 182 intersecting genes. **C** Univariate Cox analysis was performed on 134 intersecting genes in PanCK-expressing regions.**D** Survival analysis was conducted on prognostic metabolic genes in PanCK-expressing regions. **E** ROC curves were used to predict the survival rates of breast cancer patients. For the Kaplan Meier curves, the p values were calculated using the log-rank test. **Table S1.** Characteristics of breast cancer patients (n=125).

## Data Availability

The original contributions presented in the study are included in the article. Further inquiries can be directed to the corresponding authors.
