# Supplementary material for: Identifying prognostic markers in spatially heterogeneous breast cancer microenvironment
Source: J Transl Med. 2023 Aug 29;21:580. doi: 10.1186/s12967-023-04395-x (PMC10463390; doi:10.1186/s12967-023-04395-x)
Supplement: Supplementary file 1 — Additional file 1: Figure S1. Survival analysis of prognostic genes.A Survival analysis of genes in the turquoise module in tumor cell enriched regions. B Survival analysis of genes in the blue module in immune cell enriched regions. Figure S2. Differential expression of immune checkpoint-related genes among the three regions. Data shows the expression of immune checkpoint-related genes in tumor cell (PanCK), immune cell (CD45), and normal epithelial cells enriched regions (NC). Figure S3. Analysis of ER-related genes. A GSVA enrichment analysis of PanCK-expressing and CD45-expressing regions. B There were 944 metabolic genes, 3515 differentially expressed genes (PanCK-expressing regions vs. CD45-expressing regions), and 182 intersecting genes. C Univariate Cox analysis was performed on 134 intersecting genes in PanCK-expressing regions.D Survival analysis was conducted on prognostic metabolic genes in PanCK-expressing regions. E ROC curves were used to predict the survival rates of breast cancer patients. For the Kaplan Meier curves, the p values were calculated using the log-rank test. Table S1. Characteristics of breast cancer patients (n=125). [file 12967_2023_4395_MOESM1_ESM.pdf]

Fig. S1

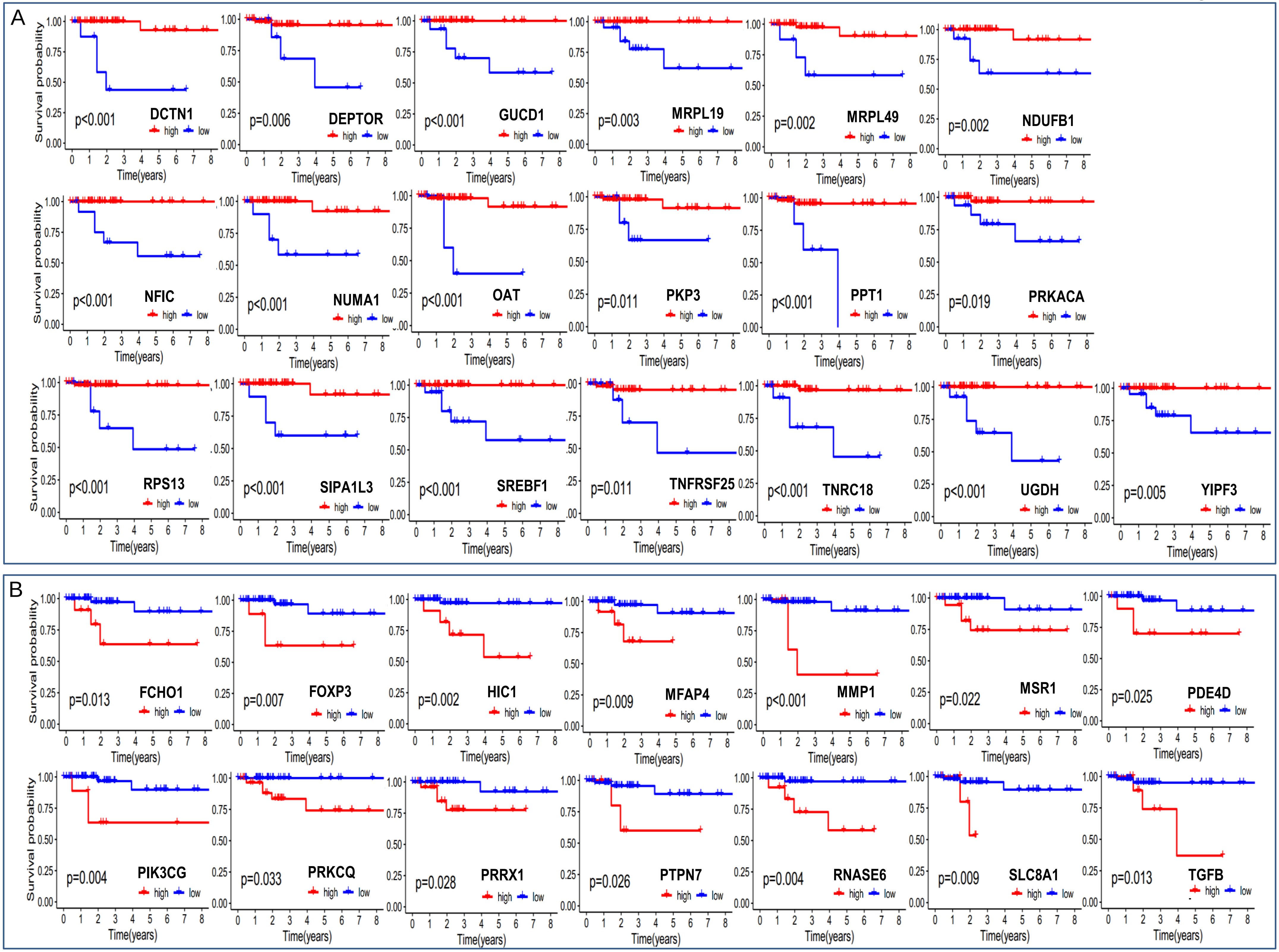

A
DCTN1 p<0.001
DEPTOR p=0.006
GUCD1 p<0.001
MRPL19 p=0.003
MRPL49 p=0.002
NDUFB1 p=0.002
NFIC p<0.001
NUMA1 p<0.001
OAT p<0.001
PKP3 p=0.011
PPT1 p<0.001
PRKACA p=0.019
RPS13 p<0.001
SIPA1L3 p<0.001
SREBF1 p<0.001
TNFRSF25 p=0.011
TNRC18 p<0.001
UGDH p<0.001
YIPF3 p=0.005
B
FCHO1 p=0.013
FOXP3 p=0.007
HIC1 p=0.002
MFAP4 p=0.009
MMP1 p<0.001
MSR1 p=0.022
PDE4D p=0.025
PIK3CG p=0.004
PRKCQ p=0.033
PRRX1 p=0.028
PTPN7 p=0.026
RNASE6 p=0.004
SLC8A1 p=0.009
TGFB p=0.013
high
low
Survival probability
Time(years)

Fig. S2

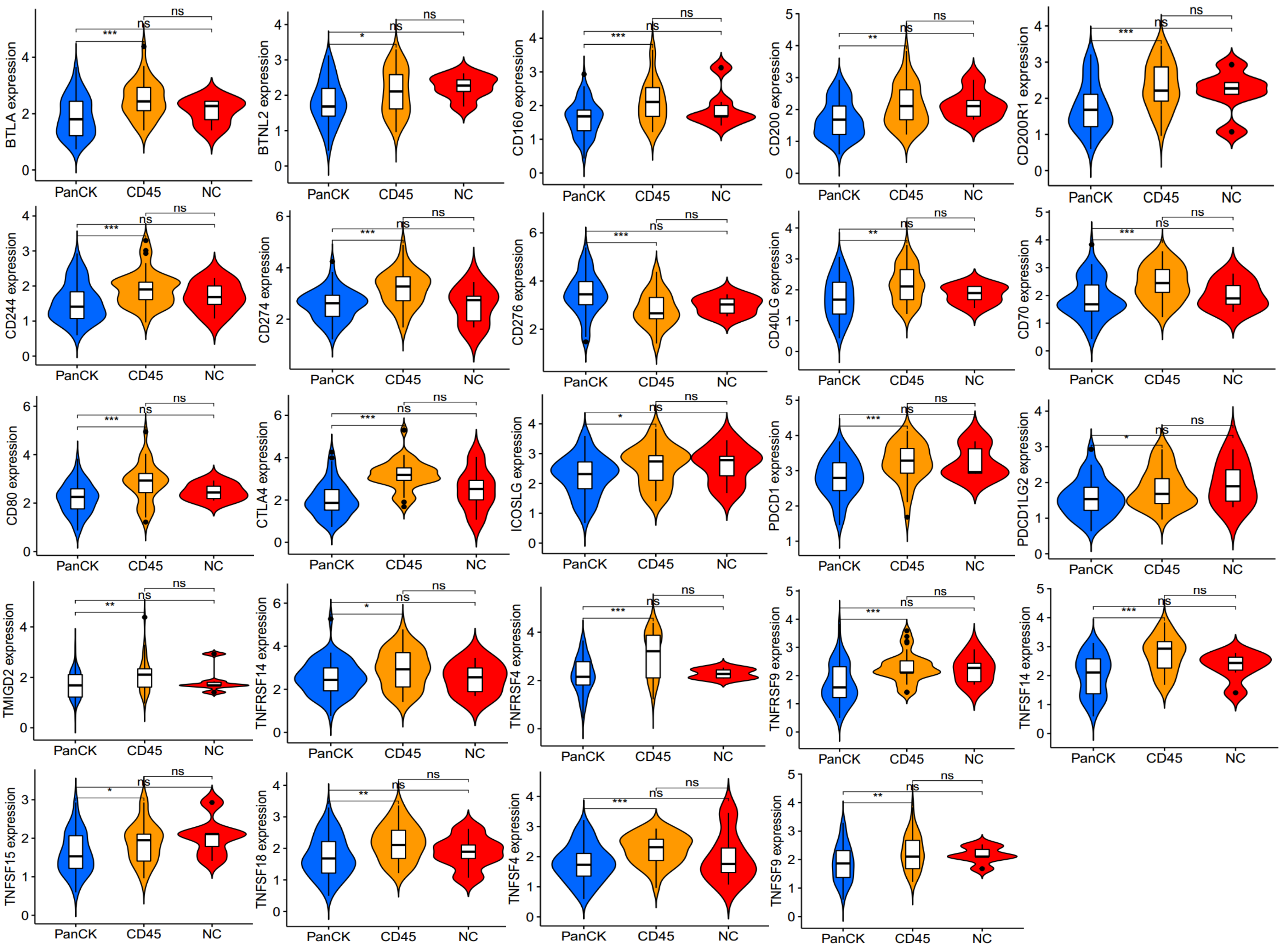
BTLA expression
BTNL2 expression
CD160 expression
CD200 expression
CD200R1 expression
CD244 expression
CD274 expression
CD276 expression
CD40LG expression
CD70 expression
CD80 expression
CTLA4 expression
ICOSLG expression
PDCD1 expression
PDCD1LG2 expression
TMIGD2 expression
TNFRSF14 expression
TNFRSF4 expression
TNFRSF9 expression
TNFSF14 expression
TNFSF15 expression
TNFSF18 expression
TNFSF4 expression
TNFSF9 expression
PanCK
CD45
NC
***
**
*
ns

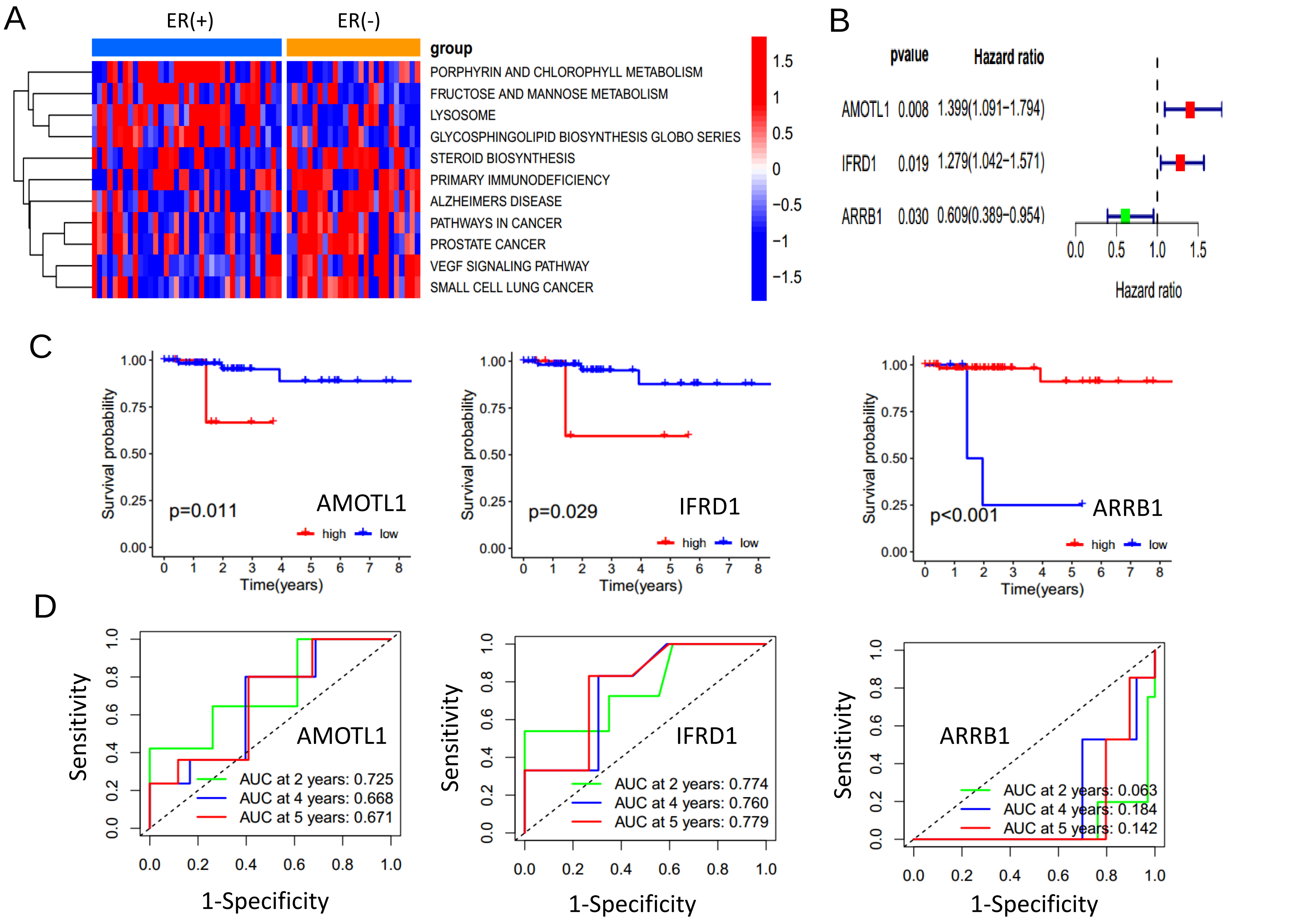
A
ER(+)
ER(-)
group
PORPHYRIN AND CHLOROPHYLL METABOLISM
FRUCTOSE AND MANNOSE METABOLISM
LYSOSOME
GLYCOSPHINGOLIPID BIOSYNTHESIS GLOBO SERIES
STEROID BIOSYNTHESIS
PRIMARY IMMUNODEFICIENCY
ALZHEIMERS DISEASE
PATHWAYS IN CANCER
PROSTATE CANCER
VEGF SIGNALING PATHWAY
SMALL CELL LUNG CANCER
1.5
1
0.5
0
−0.5
−1
−1.5
B
pvalue
Hazard ratio
AMOTL1 0.008 1.399(1.091−1.794)
IFRD1 0.019 1.279(1.042−1.571)
ARRB1 0.030 0.609(0.389−0.954)
0.0
0.5
1.0
1.5
Hazard ratio
C
Survival probability
1.00
0.75
0.50
0.25
0.00
Time(years)
p=0.011
AMOTL1
p=0.029
IFRD1
p<0.001
ARRB1
high
low
D
Sensitivity
1-Specificity
AMOTL1
AUC at 2 years: 0.725
AUC at 4 years: 0.668
AUC at 5 years: 0.671
IFRD1
AUC at 2 years: 0.774
AUC at 4 years: 0.760
AUC at 5 years: 0.779
ARRB1
AUC at 2 years: 0.063
AUC at 4 years: 0.184
AUC at 5 years: 0.142

Table S1. Characteristics of breast cancer patients (n=125)

Variable		Number
Age		
	<60	107(85.6%)
	≥60	18(14.4%)
Tumor Size		
	T1	30(24.0%)
	T2	69(55.2%)
	T3&T4	26(20.8%)
Lymph node metastasis		
	Yes	69(55.2%)
	No	56(44.8%)
Distant metastasis		
	Yes	26(20.8%)
	No	99(79.2%)
ER		
	Negative	48(38.4%)
	Positive	77(61.6%)
PR		
	Negative	54(43.2%)
	Positive	71(56.8%)
HER2		
	Negative	59(47.2%)
	Positive	66(52.8%)
